# Distribution of Misfolded Prion Protein Seeding Activity Alone Does Not Predict Regions of Neurodegeneration

**DOI:** 10.1371/journal.pbio.1002579

**Published:** 2016-11-23

**Authors:** James Alibhai, Richard A. Blanco, Marcelo A. Barria, Pedro Piccardo, Byron Caughey, V. Hugh Perry, Tom C. Freeman, Jean C. Manson

**Affiliations:** 1 The Roslin Institute and Royal (Dick) School of Veterinary Studies, University of Edinburgh, Edinburgh, United Kingdom; 2 The National CJD Research and Surveillance Unit, Centre for Clinical Brain Sciences, Western General Hospital, University of Edinburgh, Edinburgh, United Kingdom; 3 Laboratory of Persistent Viral Diseases, National Institute for Allergy and Infectious Diseases, National Institutes of Health, Rocky Mountain Laboratories, Hamilton, Montana, United States of America; 4 Centre for Biological Sciences, University of Southampton, Southampton, United Kingdom; University College London, UNITED KINGDOM

## Abstract

Protein misfolding is common across many neurodegenerative diseases, with misfolded proteins acting as seeds for "prion-like" conversion of normally folded protein to abnormal conformations. A central hypothesis is that misfolded protein accumulation, spread, and distribution are restricted to specific neuronal populations of the central nervous system and thus predict regions of neurodegeneration. We examined this hypothesis using a highly sensitive assay system for detection of misfolded protein seeds in a murine model of prion disease. Misfolded prion protein (PrP) seeds were observed widespread throughout the brain, accumulating in all brain regions examined irrespective of neurodegeneration. Importantly, neither time of exposure nor amount of misfolded protein seeds present determined regions of neurodegeneration. We further demonstrate two distinct microglia responses in prion-infected brains: a novel homeostatic response in all regions and an innate immune response restricted to sites of neurodegeneration. Therefore, accumulation of misfolded prion protein alone does not define targeting of neurodegeneration, which instead results only when misfolded prion protein accompanies a specific innate immune response.

## Abstract 2

The distribution of misfolded prion protein seeding activity alone does not predict regions of neurodegeneration in prion disease; rather, a complex microglial response appears to determine selective vulnerability and provides new strategies for therapy.

## Author Summary

Normal brain function requires tight regulation of protein folding; when this goes wrong, proteins can fold into abnormal conformations, which have severe impacts on brain performance, leading to conditions like dementia. Previous studies show that abnormally folded proteins are found in restricted parts of the brain, and neuronal cells in these specific brain regions have been shown to undergo degeneration. Recent technological advances have enhanced the detection of abnormally folded prion protein (PrP) during disease; we used these technologies to test whether distribution of abnormally folded proteins is indeed restricted to regions of the brain undergoing degeneration. Surprisingly, we observed abnormally folded proteins throughout the brain, demonstrating that these proteins can accumulate in parts of the brain that do not show degeneration. Thus, the distribution of abnormally folded protein, by itself, is not sufficient for neuronal degeneration. In addition, we found that microglia (one of the nonneuronal cell types in the brain) change their response during prion disease in two different ways; one response is associated with resilient brain regions, and the second, an inflammatory response is associated with regions susceptible to degeneration. Thus, the microglial response appears to be important in determining the outcome of disease.

## Introduction

Many chronic neurodegenerative diseases, such as Alzheimer disease, Parkinson disease and Transmissible Spongiform Encephalopathies (TSE) or prion diseases, are characterised by accumulation of misfolded proteins [1]. The appearance of detectable misfolded proteins and their relationship to neurodegeneration have been the major focus for defining disease mechanisms. However, it remains unclear what precise role misfolded proteins have in disease pathogenesis.

The prion diseases provide valuable model systems to examine this relationship and define mechanisms of neurodegenerative disease. The host protein is an absolute requirement for disease because, in the absence of prion protein (PrP), mice have been demonstrated to be resistant to disease [2,3]. Furthermore, PrP^-/-^ mice that have PrP^+/+^ tissue grafts into the brain have demonstrated deterioration of PrP^+/+^ graft tissue but preservation of host PrP^-/-^ neurons when experimentally infected with a prion disease [4]. At clinical stages, misfolded protein aggregates are usually detected in brain regions undergoing overt neurodegeneration. The accumulation and aggregation of misfolded proteins precede detectable neurodegeneration [5–9]. Recent studies have suggested that the spread of a number of different misfolded protein species between distinct brain regions occurs in a “prion-like” mechanism and is thought to determine specific brain regions that undergo neurodegeneration (reviewed in [10,11]). For example, in Alzheimer disease, the initial detection of tau-neurofibrillary tangles in the locus correleus and entorhinal cortex spreads in a pattern resembling interconnected brain regions [12], which correlates with cognitive decline in patients. Initial detection of Amyloid-beta (Aβ) is in the neocortex before spreading to allocortex and subsequently to subcortical regions in a pattern that broadly corresponds to anatomical connections. These studies can lead to the conclusion that accumulation, spread, and distribution of misfolded proteins predict regions that ultimately undergo neurodegeneration and thus define disease outcome. However, a number of findings question the direct relationship between protein misfolding and neurodegeneration. For example, misfolded PrP accumulates in the brain, in some situations unaccompanied by other typical neuropathological changes or any clinical signs of disease [13–16]. This relationship is not clear in the other protein misfolding diseases because the accumulation of Aβ as amyloid plaques, for example, has been detected in the brains of cognitively normal, aged individuals [17]. Furthermore, the detection of Aβ in Alzheimer disease patients does not always correspond to the anatomical regions accumulating neurofibrillary tangle lesions, which, as mentioned above, correlate strongly with cognitive decline [12]. Taken together, this raises the question as to the relationship between the appearance of misfolded protein aggregates and neurodegeneration.

Exquisitely sensitive methods have been developed for detection of misfolded PrP [18–20]. These methods test whether samples contain misfolded PrP isoforms, defined by their ability to convert recombinant PrP to abnormal isoforms. The products are detectable using fluorescent amyloid fibril-binding compounds, such as thioflavin-T (ThT) [18,19], therefore testing the capability of a sample to act as a “prion seed.” This therefore represents the detection of only those misfolded PrP isoforms that can seed the conversion of normally folded PrP to misfolded isoforms that may be just a portion of the total misfolded PrP present. Although seeding activity is unlikely to be the only important characteristic of pathological forms of PrP, it is an important feature in as much as it reflects the fundamental principle of the self-propagating potential of prions. The increased sensitivity of this assay, termed the real-time quaking-induced conversion (RT-QuIC) assay, allows a novel approach to study the role of misfolded protein, which can act as seeds, in relation to microglial and astrocytic responses and neurodegeneration. Moreover, the prion models allow precise time course studies to be conducted to assess the presence of misfolded protein in specific regions of the brain from initiation of disease through to neurodegeneration.

We have used the RT-QuIC assay system to examine the distribution of prion seeds in selected regions of the brain in a murine model of prion disease and compared this with the spread of misfolded PrP using other detection systems such as immunohistochemistry (IHC), neurodegeneration, and glial cell responses. We have established that the misfolded protein alone is insufficient for neurodegeneration, and a complex and heterogeneous microglia response is associated with disease.

## Results

### Misfolded PrP, Detectable Using IHC, Is Targeted to Specific Brain Regions

In this study, the GSS/101LL model of prion disease was used [14]. This involves an intracerebral (i.c.) inoculation of a human Gerstmann-Sträussler-Scheinker (GSS) brain homogenate into mice that have a proline to leucine alteration at codon 101 of the murine PrP—henceforth termed GSS/101LL. For controls, a normal brain homogenate (NBH) was inoculated i.c. into age-matched mice (NBH/101LL). Animals were assessed daily for 2 wk by animal staff independent to this study after inoculum injection to ensure condition and health of mice did not deteriorate. The important feature of the GSS/101LL model for this study is the distinct and restricted regions of pathology in the brain at the terminal stage of disease at 291 ± 5.3 days post inoculation (dpi). To define the earliest detectable accumulation of misfolded PrP and its relationship to neurodegeneration, serial sections were taken throughout the brain of GSS/101LL and NBH/101LL mice at several points within the incubation period: 150, 200, 220, and 240 dpi, and terminal stage (291 ± 5.3 dpi).

**Fig 1:**
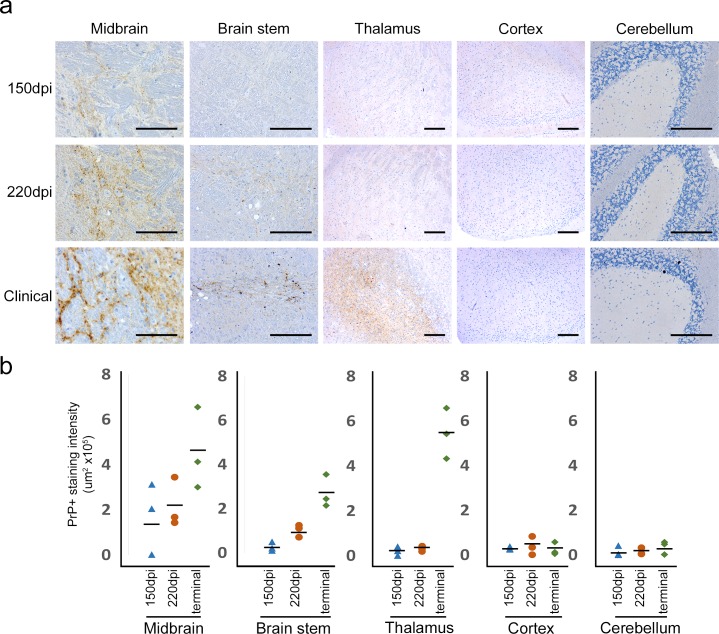
Detection of misfolded PrP using IHC at different time-points in different brain regions. (a) At 150 dpi, small quantities of fine-punctate misfolded PrP deposits can be detected in the midbrain. This positive staining could be observed in five of twelve GSS/101LL mice tested, but no staining was observed in any NBH/101LL animal in any brain region (*n* = 12). At 220 dpi, fine-punctate misfolded PrP deposits were detectable in both the midbrain and brain stem, which was observed in four of six GSS/101LL mice tested, but no staining was observed in any NBH/101LL animal in any brain region (*n* = 6). At clinical onset of disease (291.1 ± 5.3 dpi), misfolded PrP staining could be observed in midbrain, brain stem, and the thalamus but not in cortex or cerebellum in GSS/101LL mice. This staining pattern was observed in all mice tested at this stage (*n* = 9), whereas no staining was observed in any NBH/101LL animal in any brain region tested (*n* = 4). Scale bars: midbrain = 100 μm, brain stem, thalamus, cortex, and cerebellum = 200 μm. (b) Quantification of PrP+ staining intensity. The levels of PrP+ staining are originally high in the midbrain, but at later time-points in other brain regions, such as brain stem and thalamus, the levels of PrP+ staining increase to comparable levels to that of the midbrain. In cortex and cerebellum, no change in PrP+ staining was observed. Quantitation was performed using colour deconvolution plug-in to Image-J software.

Granular deposits of misfolded PrP were detected in the midbrain at 150 dpi by IHC (Fig 1). Specifically, the staining observed is associated with the interpeduncular nuclei and substantia nigra, pars compacta (SNc). No staining could be observed in any other brain region (Fig 1); therefore, the initial IHC-detectable misfolded PrP targets specific midbrain nuclei. At 200 dpi, IHC-detectable misfolded PrP remained restricted only to midbrain nuclei. At later time-points (>220 dpi), additional IHC-detectable PrP could also be detected in brain stem regions such as the medial and dorsal raphe nuclei in GSS/101LL animals. No IHC-detectable PrP was observed in any other brain region at this time-point. At clinical onset of disease, IHC detection of misfolded PrP could be observed restricted to specific neuronal populations in three major brain regions: the midbrain, brain stem, and thalamus (Fig 1). No IHC-detectable misfolded PrP was observed in other brain regions.

### Widespread Distribution of Prion Seeds

**Fig 2:**
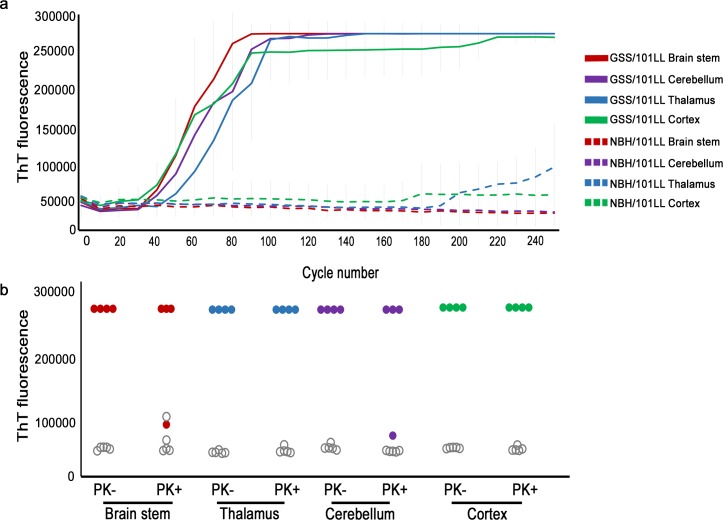
RT-QuIC shows widespread detection of misfolded prion seeds beyond levels detected using IHC. (a) ThT fluorescence readout over time during the RT-QuIC assay. Each solid line represents a GSS/101LL brain region, whereas each dotted line represents a NBH/101LL brain region. These data are comprised of the averages from triplicate RT-QuIC reactions from three separate repeat experiments collected from four separate GSS/101LL dissected brains and five NBH/101LL dissected brains all at the terminal stage of disease (291.1 ± 5.3 dpi). The different brain regions are colour coded to illustrate the brain stem (red), thalamus (blue), cerebellum (purple), and cortex (green) for both the GSS/101LL and NBH/101LL samples. (b) RT-QuIC ThT fluorescence readout at 48 h (respective to cycle 190 in the RT-QuIC assay) using samples with or without PK exposure. ThT fluorescence increases are observed in each PK-exposed brain region of GSS/101LL mice (*n* = 4), showing that the misfolded PrP responsible for the seeding event has obtained a PK-resistant conformation in all GSS/101LL brain regions. No increase in ThT fluorescence was observed in NBH/101LL control brain regions (*n* = 5), demonstrating the specificity of prion seeding ability in GSS/101LL brain regions. GSS/101LL samples are presented as red (brain stem), blue (thalamus), purple (cerebellum), or green (cortex), and region-matched NBH/101LL controls are plotted in the same columns as grey open dots. These data are comprised of triplicate RT-QuIC reactions for each brain region of each animal tested.

We hypothesised that misfolded prion seeds would also be restricted to the specific brain regions associated with IHC-detectable misfolded PrP and that these brain regions would specifically undergo neurodegeneration, whereas those with no immunopositive PrP deposits would contain no prion seeds and remain free of neurodegeneration. In order to test this hypothesis, four brain regions were assessed for the presence of prion seeds as defined by their ability to act as seeds in the RT-QuIC assay and generate a ThT-positive signal. These were two IHC-positive regions the brain stem (between Bregma -6 to -8) and thalamus (between Bregma -1 to -3) and two IHC-negative regions the cerebellum (between Bregma -6 to -8) and cerebral cortex (between Bregma -1 to -3) (henceforth referred to as cerebellum, brain stem, thalamus, and cortex). All of these brain regions from GSS/101LL mice, when used in the RT-QuIC assay, elicited an increased ThT fluorescence not observed in uninfected NBH controls (Fig 2A). Thus, the increased level of fibril formation was specific to prion infection and demonstrated the presence of prion seeds in each brain region tested. To assess whether the prion seeds detected in these brain regions represented a protease-resistant conformational rearrangement of PrP [21], proteinase K (PK) digestion was performed on all samples of brain regions prior to their inclusion in the RT-QuIC assay. In all regions from prion-infected brains exposed to PK, prion seeds remained detectable, but PK-resistant prion seeds were not observed in any region of age-matched uninfected NBH controls (Fig 2B). To confirm the widespread appearance of prion seeds, we have tested these same brain regions that gave positive RT-QuIC detection in a different assay: the protein misfolded cyclic amplification (PMCA) assay [22]. We show that, similar to RT-QuIC, all brain regions from GSS/101LL tested were capable of seeding the PMCA reaction (S1 Fig), conclusively demonstrating the widespread accumulation of misfolded PrP, which can act as prion seeds in all brain regions tested in GSS/101LL mice.

### Prion Seed Accumulation Is Not Always Linked with Neurodegeneration

**Fig 3:**
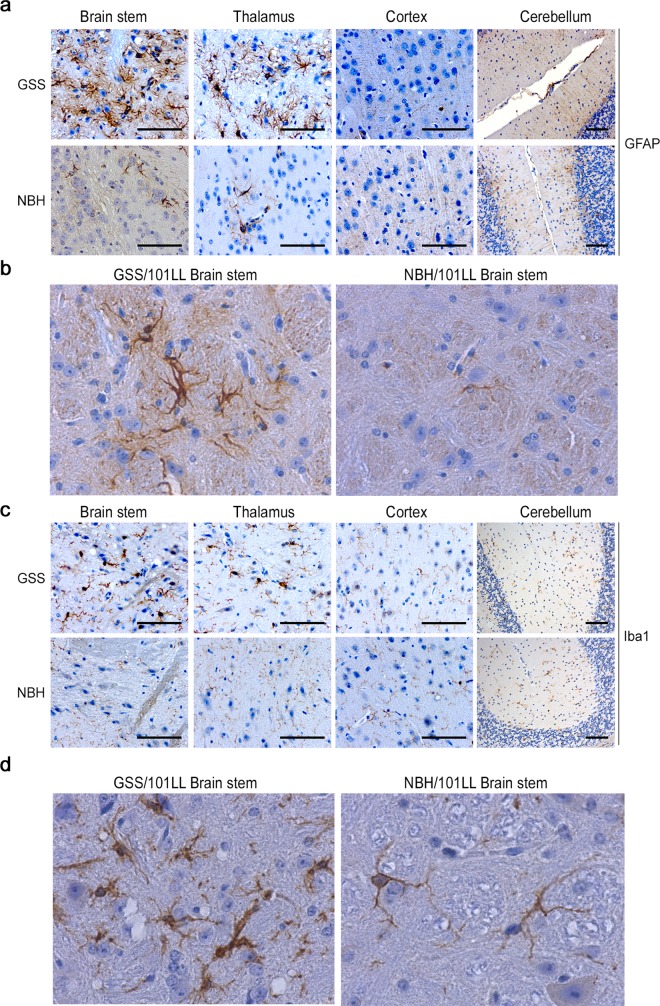
Morphological glial cell responses are restricted to specific brain regions. (a) Severe astrogliosis is observed in the brain stem and thalamus of GSS/101LL but is not detected in NBH/101LL age-matched controls or in the cortex or cerebellum of GSS/101LL mice. (b) High magnification image demonstrating the change in astrocyte expression of GFAP in GSS/101LL mice compared to equivalent NBH/101LL brain regions. (c) A distinct change in cell morphology to that of a hypertrophied cell body and short thick processes could be observed in Iba1+ cells, indicative of activated microglia, in GSS/101LL brain stem and thalamus. No change in cell morphology was observed in either NBH/101LL age- and region-matched control samples or in GSS/101LL cortex and cerebellum samples. (d) High magnification image to highlight the shortening and thickening of microglial processes, a characteristic common to morphologically activated microglia. These findings are observed consistently across all animals tested; GSS/101LL (*n* = 9), NBH/101LL (*n* = 4). Scale bars = 100 μm.

We performed an IHC analysis on the four regions from the prion-infected brains that were positive for prion seeds using the RT-QuIC (Fig 2; brain stem, thalamus, cortex, and cerebellum). Previous studies have demonstrated morphological changes associated with microglial activation and astrogliosis as valuable histological markers for early pathology, as both occur early in the course of disease before other early pathological changes as observed via histology, such as synaptic degeneration [23–27]. At clinical disease stage, we observe activated microglial and astrocyte glial cell responses. These are characterised by an up-regulation of glial-fibrillary acidic protein (GFAP), indicative of reactive astrocytes, or the hypertrophy of microglial cell bodies and thickening of microglial cell processes detected with Ionised calcium-binding adapter molecule 1 (Iba1) indicative of activated microglia. These glial cell responses were observed specifically restricted to brain stem and thalamus in GSS/101LL animals (Fig 3) but were not seen in cortex or cerebellum of GSS/101LL animals or in any NBH/101LL brain region (Fig 3).

**Fig 4:**
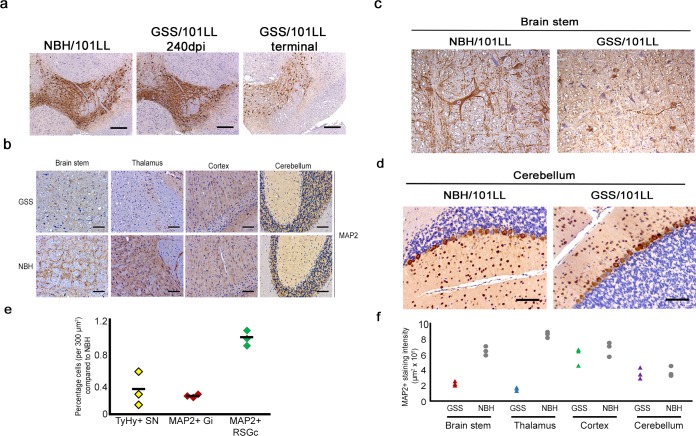
Changes in neuronal markers demonstrate specific neuronal populations targeted to certain brain regions. (a) Tyrosine hydroxylase staining of midbrain neurons. No visible change in staining pattern could be observed in any GSS/101LL animal tested at 240 dpi (*n* = 6) compared to age- and region-matched NBH/101LL controls (*n* = 6). A marked loss of staining pattern is observed in the midbrain neurons upon clinical onset (291.1 ± 5.3 dpi), indicative of a loss of tyrosine hydroxylase neurons upon clinical onset of disease. Scale bar = 200 μm. (b) MAP2 staining in brain stem has marked loss of MAP2 cell–associated staining compared to NBH/101LL brain stem age-matched control. Overall, levels of MAP2 staining are visibly lost in the ventral-medial parts of the thalamus compared to region- and age-matched NBH/101LL controls. No change could be observed in the staining pattern of MAP2 in any part of the cortex or cerebellum compared to region- and age-matched NBH/101LL controls. These findings are observed consistently across all animals tested; GSS/101LL (*n* = 9), NBH/101LL (*n* = 4). Scale bars = 100 μm. (c) Higher magnification examples of MAP2 neurons lost in the gigantoreticular nuclei of the brain stem but no loss of neurons evidenced in the cortex. (d) Parvalbumin staining of Purkinje cells of the cerebellum at clinical stages of disease in GSS/101LL animals (291.1 ± 5.3 dpi; *n* = 3) compared to age-matched NBH/101LL controls (*n* = 3). Scale bars = 100 μm. (e) Neuronal cell counts of substantia nigra (SN) neurons of the midbrain, gigantocellular reticular nuclei (Gi) of the brain stem, and retrosplenial granular region (RSGc) of the cortex from three representative animals. Cells counted based upon the number of cells showing positive staining for either tyrosine hydroxylase (TyHy+) in the SN or MAP2 in the Gi and RSGc. (f) Quantification of MAP2+ staining intensity from three representative animals showing a loss of MAP2 staining in brain stem and the thalamus but no change in the cortex or cerebellum. Quantification of staining was performed using colour deconvolution plug-in of Image-J software.

Midbrain neurons were assessed by their expression pattern of tyrosine hydroxylase, and no change in its staining pattern could be detected until clinical onset of disease (Fig 4A), at which point significant cell loss was observed (Fig 4E). Cell loss was also quantified in specific neuronal populations in the brain stem and cortex and showed substantial cell loss in the brain stem but not in the cortex (Fig 4E). Brain stem, thalamus, cortex, and cerebellum neurons were assessed using antibodies against microtubule associated protein 2 (MAP2), isoforms a+b. No change in MAP2 staining could be observed in cerebellum or cortex neurons at clinical stages of disease (Fig 4B and 4F). Substantial changes in MAP2 staining were observed in brain stem nuclei, such as the gigantocellular reticular nuclei (Fig 4B, 4C and 4F). This encompassed a general loss in precise cell body–associated staining compared to region- and age-matched NBH/101LL controls, which is indicative of neuronal degeneration in GSS/101LL brain stem. A reduction of MAP2 staining associated with dendritic processes was observed in the thalamus at clinical stages of disease, which was particularly prevalent in the ventral-medial parts of the thalamus (Fig 4B and 4F). To further assess specific neuronal populations of the cerebellum, antibodies specific to the calcium-binding protein parvalbumin were used. Parvalbumin is highly expressed in Purkinje and stellate and basket neurons of the cerebellum and has been shown to be lost as a result of pathology in the cerebellum [28]. No change in the pattern of staining or intensity was observed in the GSS/101LL cerebellum compared to uninfected controls, even at clinical stages of disease (Fig 4D).

Finally, we analysed the presence of spongiform vacuolation of the neuronal parenchyma, which is a pathology commonly associated with prion disease and is quantified using well-established protocols, which we followed in our study [29]. We observed very low levels of vacuolation in most areas of the brain that were analysed, with the highest levels of vacuolation being observed in the medulla (brain stem) and negligible levels found in cerebellum or cortical regions (S2 Fig). Taken together, these data show that neurodegeneration and morphological glial cell responses are restricted to the brain stem and thalamus, whereas cortex and cerebellum appear to remain pathologically “unaffected,” even at clinical stages of disease.

### Absence of Overt Neurodegeneration Is Not Due to Time of Exposure or Differing Levels of Prion Seeds

**Fig 5:**
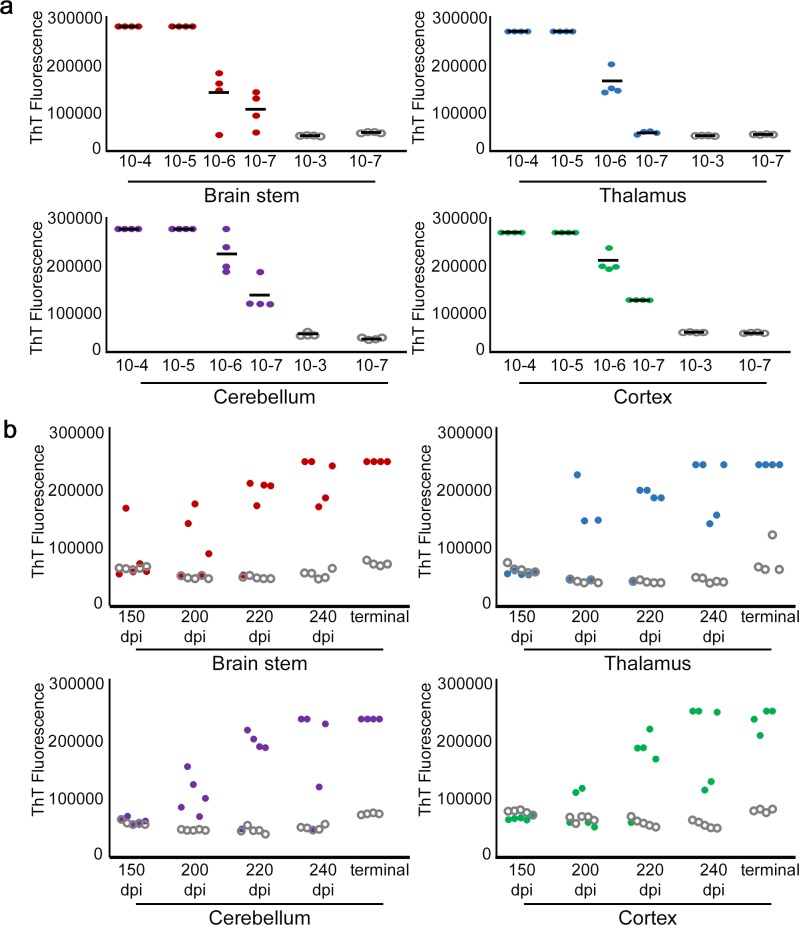
Neither quantity nor time of exposure of misfolded prion seeds are responsible for restricted neurodegeneration. (a) RT-QuIC titration of brain region homogenates (*n* = 4) shows that all brain regions dilute to a concentration of at least 0.001% (w/v) of the wet sample weight (10^−5^). Data shown are the average ThT fluorescence levels after 60 h incubation in RT-QuIC of triplicate RT-QuIC reactions. Grey open dots represent NBH/101LL region-matched controls. (b) RT-QuIC run on GSS/101LL brain regions at several time-points throughout the incubation period. Each sample was run at a concentration of 0.1% (w/v) of the wet sample weight (10^−3^) for *n* = 5 GSS/101LL and NBH/101LL mice at each time-point, with exception of clinical (terminal) stage of disease (*n* = 4) and run in triplicate RT-QuIC reactions. Grey open dots demonstrate average ThT fluorescence of NBH controls (*n* = 5) at each time-point. Data shown are the ThT fluorescence levels after 48 h incubation in RT-QuIC.

Several arguments could be made to explain the apparent lack of neurodegeneration in regions exposed to prion seeds, such as the following: (i) the amount of prion seeds may vary between regions undergoing neurodegeneration and regions that appear unaffected; (ii) tissue undergoing neurodegeneration may have been exposed to prion seeds for comparatively longer periods of time than regions that remain unaffected; or (iii) prion seeds are necessary, but not sufficient, for neurodegeneration. To address scenario (i), we ran the RT-QuIC assay with decreasing concentrations of homogenate from each brain region to determine at what dilution detection of prion seeds is lost. Prion seeds were observed to support generation of a ThT signal at a concentration of at least 10^−5^ of original brain homogenate, or 0.001% (w/v) of original sample mass (Fig 5A). Thalamus, cerebellum, and cortex all showed an increased ThT fluorescence at a concentration of 10^−6^ (0.0001% w/v of original sample mass). These data show no relationship between quantity of detectable prion seeds and neurodegeneration, as defined in Figs 3 and 4. To address scenario (ii), we tested for prion seeds at all time-points used in this study (150, 200, 220, and 240 dpi and terminal illness). At 200 dpi onwards, an increased ThT fluorescence was observed in brain stem and thalamus, which eventually succumb to neurodegeneration (Fig 5B), and in cerebellum, which does not undergo neurodegeneration (Fig 5B). This is reaffirmed at later time-points, with increased ThT fluorescence also observed in GSS/101LL cortex samples (Fig 5B). The data show that detectable prion seeds become widespread at relatively early but specific stages of disease progression but appear not to be associated with resilience or susceptibility of a particular brain region to neurodegeneration.

### Transcriptional Response to Accumulation of Prion Seeds in the Presence or Absence of Neurodegeneration

**Fig 6:**
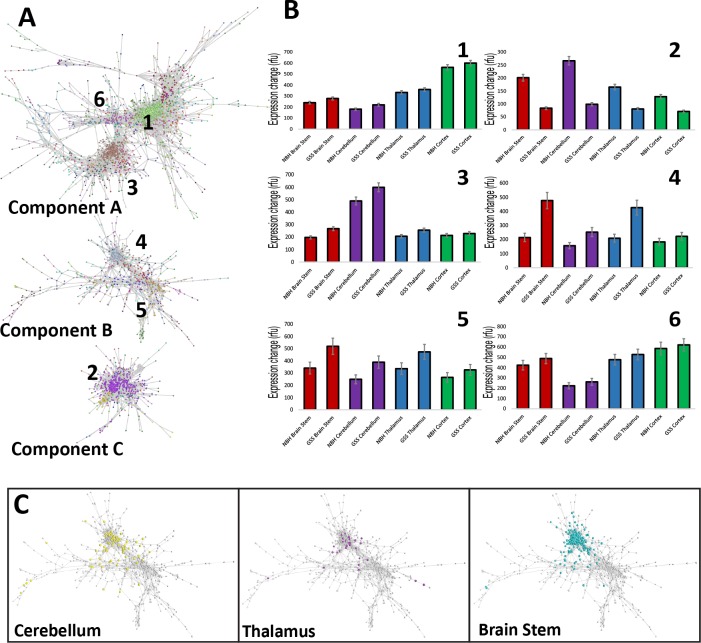
All GSS/101LL brain regions show disease-associated gene expression changes. (A) Biolayout *Express*^3D^ graph showing a spatial representation of genes orientated according to their correlation to one another. Three major and separate components of highly correlated genes were formed using this software, which we term components A–C. This structure was used by the Markov clustering algorithm to divide the graph into clusters of co-expressed transcripts, which are shown in the graph as different colours. Representative clusters, shown as numbers 1–6, are shown on the graph, which highlights the expression differences found between each of the three major components identified. These can be viewed as average gene expression as bar graphs (B). (C) Filtered gene lists from the GSS/101LL cerebellum, thalamus, and brain stem are overlaid onto the original graph. Shown here are the filtered genes highlighted in yellow (cerebellum), purple (thalamus), or turquoise (brain stem) as part of component B of the main graph shown in (A). The highlighted genes in each brain region were observed predominantly within the same part of the graph rather than segregating into distinct groups, demonstrating that these differentially expressed genes between brain regions were highly correlated.

A transcriptomics analysis was performed on the four brain regions to help define host responses to misfolded PrP in the presence or absence of neurodegeneration. Mice were separated into three groups, with two mice per-group; RNA was extracted from individual brain regions, and each group was pooled together. To interpret the microarray data, we used the network analysis tool BioLayout *Express*^3D^ [30] in combination with statistical methods. Following data normalisation, the software calculates a pairwise Pearson correlation matrix comparing the expression profile of each transcript represented on the array to the expression profile of all other transcripts. All correlations above a user-defined threshold are used to construct a network graph visualised in 3-D space. When groups of genes are similarly expressed, they are tightly correlated and give rise to highly connected cliques within the network. This structure is used by the Markov clustering algorithm to divide the graph into clusters of co-expressed transcripts [31]. The network topography of our data produced three groups of genes (components A–C), which do not share direct correlation between one another. Within each component, there are several distinct clusters of highly correlated genes (Fig 6A). The major clusters of genes in component A (e.g., clusters 1, 3, and 6) show differential gene expression between brain regions but have no obvious relationship to disease (Fig 6) and therefore were not examined further. However, components B and C both contain disease-specific clusters (Fig 6). Component B contained the largest group of disease-associated genes, and further analysis concentrated on this group.

### Up-Regulation of a Large Group of Genes across All Brain Regions Can Be Attributed to a Microglial Response

**Fig 7:**
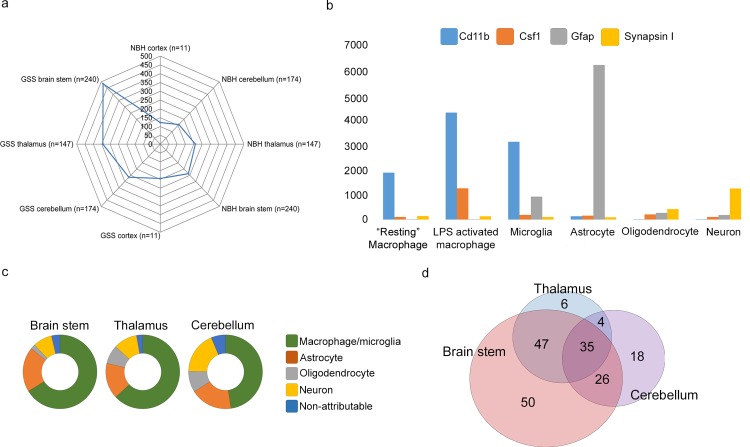
Disease-associated gene expression changes can be predominantly attributed to microglia in all GSS/101LL brain regions tested. (a) Spider graph representation of component B genes after filtering. Up-regulation of genes in all GSS/101LL brain regions, but particularly increased in GSS/101LL brain stem and thalamus compared to GSS/101LL cerebellum and cortex. N = number of genes present after data are filtered that constitute the average intensity value. The number of genes represented is highest in GSS/101LL brain stem but lowest in GSS/101LL cortex. (b) Gene expression can be attributed to specific cell types when overlaid onto previous microarray datasets. These data show a simplified version to demonstrate how different genes that are known to have selective expression in specific cell types in vivo can be attributed to their expected cell type. For example, Cd11b is a gene generally regarded as a pan-macrophage marker, and hence we show the increased expression of this gene in macrophage/microglial cell populations compared to other cell types. Colony-stimulating factor 1 (Csf1) is a gene that is up-regulated during immune cell activation, shown here by its increased expression in lipopolysaccharide-activated macrophages. Gfap is a gene expressed highly in astrocytes, and, indeed, we show the high and specific expression of Gfap in astrocytes in this dataset. Finally, synapsin I is a synaptic-specific protein and therefore will most commonly be expressed in neurons, as is shown here. (c) Attribution of genes that are identified in component b to their respective cell type shows that a majority of genes that are identified in component b can be attributed to macrophage/microglia. (d) Representation of the macrophage/microglia gene list overlap of different brain regions tested.

Data from component B were filtered stringently by first removing transcripts not annotated to known or predicted protein coding regions of the genome. Annotated genes were then filtered by Mann–Whitney *U* test (*p* ≤ 0.05) to define significantly altered genes by comparison of GSS/101LL brain regions to their respective NBH/101LL-matched control brain regions. Furthermore, of those statistically significant genes, only those that exhibited a >1.5-fold change between control and disease were analysed further. This group of genes exhibited an increase in gene expression across all brain regions, with the greatest increases observed in regions of neurodegeneration (brain stem and thalamus) and lower but significant increases in gene expression in brain regions that do not show neurodegeneration (cerebellum and cortex) (Fig 7A). Within component B, only a few significant gene expression changes were noted in the cortex (11 genes) compared with a much larger number in the other regions, and therefore the cortex was not included in further analyses in the current study.

To determine the cellular origins of component B, the filtered gene list was overlaid onto previously published expression datasets of isolated cell populations. This included a variety of murine myeloid populations (including microglia) [32] and brain cell populations (neurons and glial cells) [33]. Using these datasets, in many cases we could attribute expression of specific genes to cell type of origin (Fig 7B). The majority of up-regulated genes in the brain stem, thalamus, and cerebellum could be attributed to cells from a myeloid lineage and therefore most likely derived from the resident microglia population (Fig 7C). Thus, the microglial response appears to be an important host response in disease, with expression changes evident in regions with and without IHC detectable glial cell changes (Fig 3A). The specific genes attributed to microglia were sorted according to gene overlap (Fig 7D), showing distinct groups of genes that have differential expression in specific brain regions.

To broaden our analysis of this pattern of distinct groups of genes, we next examined the total microarray dataset, filtered according to the same statistical and fold-change parameters outlined above (*p* ≤ 0.05; >1.5-fold change) and attributed to predicted cellular origin (S1 Table). Gene ontology (GO) enrichment of the microglial gene lists was assessed in two groups: (1) genes not directly associated with neurodegeneration (i.e., all microglial genes identified as up-regulated in the cerebellum) and (2) genes associated with neurodegeneration (i.e., all microglial genes up-regulated in brain stem and thalamus but not differentially expressed in cerebellum). Amongst the microglial genes up-regulated but not directly associated with neurodegeneration, the major GO terms relate to metabolism and regulation of homeostasis (S2 Table). This demonstrates a response of microglia, which they are known to exhibit, but has never, to our knowledge, been observed in chronic neurodegenerative diseases. In genes up-regulated in brain regions undergoing neurodegeneration, significantly enriched GO terms are related to activation of the innate immune response, complement activation, and antigen processing and presentation (S3 Table), which concurs with previous data, suggesting the innate immune response as an important and necessary component of the neurodegenerative process.

## Discussion

We have demonstrated that, during the evolution of prion disease, prion seeds are widespread, accumulating in brain regions both with and without overt neurodegeneration. We show that the quantity or time of exposure to prion seeds was not directly related to the development of neuropathology. Therefore, these pathological differences are not due to a lack of spread and accumulation of prion seeds, indicating that prion seed accumulation is not itself sufficient for neurodegeneration, at least within the lifetime of the mouse.

This extensive and widespread distribution of prion seeds has not been previously described, and the highly sensitive assay developed for the detection of misfolded PrP has allowed us to further investigate our understanding of the relationship of misfolded PrP and neurodegeneration. In this study, we performed an i.c. inoculation that results in widespread dispersion of the homogenate and its rapid degradation and clearance [34]. RT-QuIC detection of prion seeds from GSS/101LL mice was not increased over NBH/101LL age-matched controls at 150 dpi; therefore, the RT-QuIC assay is detecting specific murine replication and accumulation of misfolded protein at later time-points and not the initial human inoculum. As a result, the RT-QuIC provides a highly sensitive assay system for examining the prion seed accumulation and its interactions over the course of disease.

We show that detection of misfolded PrP using IHC is restricted to specific brain regions undergoing neurodegeneration and associated morphological glial responses. Specific brain regions, for example, the thalamus, only accumulate IHC-positive misfolded PrP at late stages of disease (291 ± 5.3 dpi), whereas prion seeds are detected three months earlier in this region (200 dpi). Some authors have asserted that misfolded protein aggregates are responsible for causing neurodegenerative diseases and that the targeting of neurodegeneration is influenced by the restricted distribution of misfolded protein between brain regions [35,36]. These studies rely on detection of misfolded proteins using techniques such as IHC or basic histology staining with silver staining (reviewed in [10]). In this study, by using an alternative and highly sensitive method for the detection of misfolded PrP, which act as prion seeds, we find the distribution of misfolded PrP seeding material does not predict the regions of neurodegeneration.

IHC has been used extensively in prion disease as a terminal marker of the pathology in the brain. Although, in time course studies, PrP IHC can be detected earlier in the disease process, we believe the more sensitive assay systems (RT-QuIC and PMCA) are capable of detecting the earlier events that ultimately lead to the IHC-detectable protein. Thus, the sensitive assays both demonstrate that the pathways of protein misfolding are activated in all brain regions. Although uncertainties remain about the exact nature and toxicity of the abnormal PrP that is detected by IHC, the same can be said of RT-QuIC and PMCA. The use of sensitive techniques has uncovered the novel finding that these pathological differences are not due to a lack of spread and accumulation of prion seeds; rather, they correlate with local cellular differences in the responses to those seeds that govern whether those seeds lead to bulk PrP accumulation and pathological lesions. Although seeding activity is not likely to be the only important characteristic of pathological forms of PrP, it is likely to be one of the most important features in as much as it reflects the self-propagating potential of prions.

The question remains as to why only specific regions of the brain succumb to neurodegeneration. Previous studies have shown that the same prion strain inoculated into different murine genetic backgrounds, which exhibit comparable quantities of detectable prion seeds, can result in different sized aggregates of misfolded protein [37]. Just as large prion particles have been shown to have lower infectivity per unit mass of PrP, i.e., specific infectivity [38], so might larger prion seed particles be expected to have lower seeding activity per mass than smaller particles. If so, then two regions could have the same seed concentration but divergent total loads of abnormal PrP. In addition, if the seeds are more clustered in one brain region than another, they would also be more likely to be detectable by IHC. If different types of aggregates are accumulating across brain regions, this is likely to impact on production and clearance of aggregates in each region, with the type of misfolded protein being the driver of the response. An alternative explanation is that different regions of the brain have differing abilities to respond to the same aggregates due to intrinsic variability in gene expression of cells present. A specific example would be the underlying differences in microglial gene expression between brain regions in healthy young and aged brains, which might underlie the specificity of microglial response during disease [39]. Thus, regional differences in protein aggregate and/or cell signalling could influence targeting of neurodegeneration.

We analysed transcriptional responses in each brain region and showed that the major response in each brain region tested could be attributed to microglia, similar to previous microarray studies on prion disease cases in human and animals [40–44]. Functional annotation of the microglial response to misfolded protein, but in the absence of neurodegeneration, revealed a large proportion of genes related to regulation of homeostasis. This highlights a disease-specific response of microglia up-regulating a core set of genes, the key function of which may be to respond to changes associated with protein misfolding. However, differential activation of the innate immune response is also observed in brain regions undergoing neurodegeneration. These data suggest at least two distinct microglial responses occurring during disease. In one, microglia respond to either the protein misfolding and/or the consequences of protein misfolding by attempting to maintain homeostasis. In the other, in regions of neurodegeneration, microglia up-regulate an innate immune response, which includes pro- and anti-inflammatory genes, complement activation, and antigen processing and presentation.

**Fig 8:**
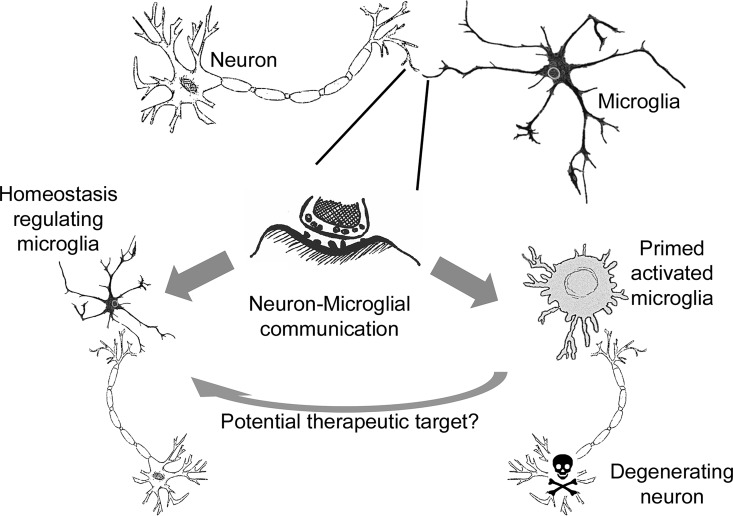
Microglial-neuron communication may define the relative resilience or susceptibility of neurons to degenerate. Microglia are known to survey the neuronal parenchyma and interact intermittently with all parts of the neuron. The accumulation of misfolded PrP, and potentially the different types of aggregates, will have an impact on the communication and interaction between neurons and microglia. As a result, we observe different microglial responses during disease as well as selective vulnerability of neurons to degenerate in specific brain regions. It remains unclear whether the physiological differences of neuronal signalling, the known gene expression differences of microglia between brain regions [37], or the different types of misfolded PrP aggregate are responsible for the different microglial activation states. Based on the associations between the different microglial activation states, the restricted neurodegeneration observed, and current knowledge of the importance of innate immune activation in defining severity of neurodegeneration, we speculate that the different microglial activation states could be defining neurodegeneration between different brain regions. This could either occur as a protective microglial response in regions showing resilience to neurodegeneration or a contributor to neurodegeneration in susceptible regions, or both. Our study highlights the need to further understand the basis for different microglial activation states, which could allow future studies to manipulate microglial responses from a primed activated state to one that regulates homeostasis and, thus, could represent a vital therapeutic target for intervention during disease.

The data presented here demonstrate that the seeding and distribution of misfolded protein are more widespread at earlier time-points than previously described. Importantly, this seeding occurs in brain regions that do not undergo neurodegeneration. We show that there is a significant host response in all brain regions examined either in the presence or absence of neurodegeneration. These responses are predominantly associated with microglial cells, and functional annotation demonstrates distinct responses from these cells between brain regions accumulating misfolded protein seeds either in the presence or absence of neurodegeneration. Therefore, microglia appear to be on the one hand attempting to restore homeostasis introduced by misfolded proteins that act as seeds, but in regions of neurodegeneration, the microglia enter into a cycle of innate immune activation. Previous studies have demonstrated that, by altering specific aspects of the innate immune response by KO of specific pro- or anti-inflammatory genes, the severity of disease can be altered [45–51]. Furthermore, by inhibiting microglial proliferation during disease, and thus reducing the number of microglia exhibiting an activated innate immune response, other studies have shown a prolongation of incubation period in prion disease [52]. Although it remains unclear what signals microglial cells are specifically responding to, specific misfolded protein isoforms or neurodegeneration, data from previous studies together with data presented here could point to a role for the activation of the innate immune response in defining severity of disease, which may contribute to the destruction of cells in specific brain regions. Therefore, manipulation of the activation state of microglia could represent a therapeutic target for suppressing neurodegeneration during disease (Fig 8). In summary, we find that the pathological differences between brain regions during chronic neurodegeneration are not due to a lack of spread and accumulation of prion seeds; rather, they correlate with local cellular differences in the responses to those seeds that govern whether those seeds lead to bulk PrP accumulation and pathological lesions. A combined approach characterising the host responses to the misfolded protein accumulation and distribution, therefore, is required to more precisely determine the relevance of particular misfolded protein species to disease outcome.

## Materials and Methods

### Ethics

All experiments were approved by the Roslin Institute Ethical Review committee and in accordance with the United Kingdom Home Office Regulations (Animals [Scientific Procedures] Act of 1986). Ethical consent for the use of human materials for research was obtained and approved by the Lothian National Health Service Board Research Ethics Committee (reference: 2000/4/157).

### Animal Husbandry

The “101LL” transgenic mouse line contains an amino acid alteration from proline to leucine at codon 101 of the 129/Ola (129/OlaHsd, Harlan, UK) murine prion gene by gene-targeting [53]. 129/Ola mice homozygous for the targeted allele were crossed and bred over multiple generations to generate progeny homozygous for the *Prnp*^P101L^ (101LL) allele, which can be used for experimental purposes. All mice were bred at the Roslin Institute under a temperature-controlled, 12 h light/12 h dark cycle. Mice were housed with wood chip bedding and a wood chew stick for environmental enrichment. Food and water were available ad libitum.

### Genotyping

To confirm the presence of the targeted allele alteration, mice were genotyped before and after studies. DNA extraction was performed using DNeasy Blood and Tissue kit (Qiagen) on ear clips. Presence of the 101LL mutation was determined by PCR analysis using a primers specific for positions 107–130 and 871–848 of the PrP gene; the 5′ primer used was (5′-ATGGCGAACCTTGGCTACTGGCTG–3′; DDBJ/EMBL/GenBank accession number M18070), and the 3′ primer used was (5′–TCATCCCACGATCAGGAAGATGAG–3′; DDBJ/EMBL/GenBank accession number M18070). PCR was set up using a Type-it mutation detection kit (Qiagen). PCR cycle conditions were as follows: 94°C for 3 min, followed by 30 cycles of 94°C for 30 sec, 62°C for 30 sec, and 72°C for 1 min. A final extension phase of 72°C for 10 min was performed, and samples were subsequently stored at 4°C. The induced alteration of proline to leucine in 101LL mice forms a DdeI restriction enzyme cut site, which allows for identification of 101LL homozygous mice. The PCR product was incubated for 3 h at 37°C with DdeI restriction enzyme (Promega) before being run on a 1.5% agarose gel (Invitrogen) and imaged.

### Prion Inoculum and Challenge

Human frontal cortex from a patient with GSS carrying the *PRNP*^-P102L^ mutation was obtained from the CJD Brain and Tissue Bank (Edinburgh Brain and Tissue Banks). The tissue was homogenised in 0.9% sterile saline (Martindale Pharmaceuticals, UK) to a 1% (w/v) homogenate, and mice were injected i.c. into the right hemisphere with 20 μL of 1% homogenate under anaesthesia. Due to the difficulty in obtaining uninfected human tissue as a negative control, an i.c. injection of 20 μL uninfected hamster brain homogenate (1% w/v) was performed instead. These protocols were used to replicate those of previous studies using this model of prion disease [14]. These form the GSS/101LL and NBH/101LL groups, respectively, which are referred to throughout the text. All animals were aged-matched and were injected between 10–14 wk of age. A time-course analysis was set up in this study, ranging from 150 dpi (GSS/101LL *n* = 12; NBH/101LL *n* = 12), 200 dpi (GSS/101LL *n* = 12; NBH/101LL *n* = 12), 220 dpi (GSS/101LL *n* = 6; NBH/101LL *n* = 6), 240 dpi (GSS/101LL *n* = 6; NBH/101LL *n* = 6), and upon clinical symptoms of disease (GSS/101LL *n* = 9; NBH/101LL *n* = 12). All animals were assigned to groups and assessed daily from around 100 dpi by an independent researcher to this study using parameters that have been previously described [54]. All mice were killed by CO_2_ asphyxiation according to Schedule 1 of the Animals (Scientific Procedures) Act of 1986.

### Preparation of Tissue for Analysis

The brains of 101LL (GSS/101LL and NBH/101LL) mice were removed and halved along the midline between the right and left brain hemispheres. The left hemisphere was flash frozen in liquid nitrogen and stored at -80°C for later use. The right hemisphere, which contains the injection site, was immersion fixed in 10% formal saline for 48 h before being exposed to 98% formic acid for 90 min to minimise the infectious titre of the sample. The tissue was subsequently re-washed in 10% formal saline for at least 24 h to remove residual formic acid. The tissue was cut into five coronal sections for vacuolation scoring [29], which encompasses nine grey matter (GM) regions (medulla, cerebellum, superior colliculus, hypothalamus, thalamus, hippocampus, septum, rerospinal cortex, and cingulate and motor cortices). Tissue was then paraffin embedded. Haematoxylin and eosin (H&E) staining of 6-μm sections were taken for vacuolation (spongiform) severity scoring, which was performed blind by a researcher independent to this study. For further pathological analysis, serial 10-μm sections were cut through the brain.

### Immunohistochemistry

Paraffin-embedded tissue was dewaxed by immersing in xylene and re-hydrated through a series of decreasing alcohol concentrations at room temperature. For PrP immunostaining, slides were immersed in citric buffer (0.1 M citric acid, 0.1 M Sodium Citrate, pH 6.4) and autoclaved at 121°C for 15 min. Slides were then cooled in running water for 5 min before immersing in 98% formic acid for 10 min. Subsequently, slides were thoroughly washed in running water for 20 min. To block for endogenous peroxidase, slides were immersed in 1% H_2_O_2_/methanol before washing in running water for 5 min followed by PBS/1% BSA wash buffer for 5 min. Sections were subsequently incubated with Normal Goat Serum (Stratech) for 20 min before application of either BH1 ([55] used at 0.02 μg/mL) or 6H4 (Prionics used at 3 μg/mL) anti-PrP antibodies. The primary antibodies were incubated overnight before washing with PBS/1% BSA wash buffer. Goat anti-mouse secondary antibody (Jackson ImmunoResearch) was applied for 1 h, washed in PBS/1% BSA, and ABC kit (Vector Laboratories) was applied for 30 min then washed. Peroxidase activity was visualised using diaminobenzidine (DAB):H_2_O_2_ and slides were counterstained in Harris’ haematoxylin.

For the immunohistochemical detection of other proteins, sections were either given no antigen-retrieval step (e.g., GFAP) or were immersed in citric buffer (pH 6.0) and autoclaved for 15 min at 121°C. The protocol replicated that of PrP detection, with exception of 10 min incubation in 98% formic acid.

### Cell Counts and DAB Quantification

Ten serial sections from three GSS/101LL and three NBH/101LL mice at clinical stage pathology from three anatomically distinct brain regions, the gigantocellular reticular (Gi) nucleus of the brain stem, the retrosplenial granular cortex (RSGc), and the SNc were cut at anatomically equivalent regions, approximately Bregma -6 mm (brain stem), Bregma -1.8 mm (cortex), and Bregma -3 mm (midbrain).

Digital images were captured at x20 magnification using a Nikon E800 bright-field microscope. A calibrated grid (100 x 100 μm) was overlaid onto each image, and the number of cell bodies was determined by counting. The total number of cells counted in GSS/101LL brain regions was then normalised to the region matched NBH/101LL controls and presented as the percentage proportion of cells present in GSS/101LL compared to NBH/101LL.

DAB quantification was performed on ten serial low-magnification images (x4) of brain stem, thalamus, cerebellum, and cortex, examining the total quantity of positive staining in each section and averaging across the serial sections. DAB quantification was performed using Image J and the colour deconvolution plug-in.

### RT-QuIC Assay

*Prnp* DNA sequences encoding Syrian hamster residues 23 to 137 followed by sheep residues 141 to 234 of the R154 Q171 polymorph (accession number AY907689) (Ham/Shp chimeric PrP) were prepared according to previous methods [20]. RT-QuIC buffer composition was as follows: 10 mM phosphate buffer (pH 7.4), 130 mM NaCl, 10 μM Thioflavin T (ThT), 10 μM EDTA, and a final concentration of 0.1 mg/mL recPrP. Ninety-eight μL of this master mix were loaded onto black 96-well clear bottom plates (Nalgene Nunc International). Correspondingly, 2 μL of diluted brain homogenate were added to each well for a final reaction volume of 100 μL. Each sample was run in triplicate, and included in each reaction were standard positive controls (79A murine scrapie prion disease at a concentration of 0.1% [w/v] of the original brain weight) and negative controls (uninfected murine brains at a concentration of 0.1% [w/v] of the original brain weight and RT-QuIC master mix–only samples). Plates were then sealed with a plate sealer film (Nalgene Nunc International). A PolarSTAR Omega (BMG Labtech) plate reader was used to incubate the samples at 42°C for 60 h with cycles of 1 min rest and 1 min 700 rpm double orbital shake. ThT fluorescence was then measured (450 nm excitation/480 emission) every 15 min during the 60 h incubation.

### PMCA Assay and Western Blot

PMCA experiments were performed following the amplification procedure described previously [56]. Briefly, aliquots of PMCA substrate, derived from transgenic mice P101L brains, were incubated with PMCA seeds in 0.2-mL PCR tubes to a final volume of 120 μL. Serial cycles of sonication and incubation were performed for 48 h at 37°C, comprising 20 sec of sonication (at an amplitude of 38, wattage: 278–299) followed by 29 min 40 sec of incubation for each cycle (Qsonica, model Q-700). Detection of PrP^res^ was assessed by PK treatment and western blotting methodology previously described [56].

### RNA Extraction

Brain stem, thalamus, cerebellum, and cortex were dissected from 6 GSS/101LL animals at terminal illness and from 6 NBH/101LL age-matched mice. The tissue was weighed then homogenised in 1 mL Trizol (Life Technologies) per 100 mg of tissue. The homogenate was thus centrifuged at 11,500x g for 10 min at 4°C. The pellet was discarded. Chloroform (0.2 mL/mL Trizol) was then added to cause phase separation, whereby protein constitutes the organic phase, DNA the interphase, and RNA the aqueous phase after a 11,500x g centrifugal step for 15 min at 4°C. The aqueous phase was transferred to fresh RNase-free tubes (Life Technologies). Isopropanol (0.5 mL/mL Trizol) was added and incubated for 10 min at room temperature. The RNA sample was then centrifuged for 10 min at 11,500x g at 4°C. The RNA pellet was washed in 75% ethanol (1 mL/mL Trizol) then centrifuged for 5 min at 8,000x g at 4°C. The RNA pellet was then resuspended in Nuclease Free dH_2_O (Life Technologies), aliquoted, and stored at -80°C.

### Microarray

RNA was extracted from brain regions of individual animals. To remove non-disease–specific inter-animal variation, each sample consisted of a pool of two animals for an individual brain region. This resulted in 24 samples. Samples were subjected to transcriptomics analyses using Mouse Gene 2.0 array run on a GeneTitan instrument (Affymetrix) by Edinburgh Genomics (www.genomics.ed.ac.uk). Data were normalised using Affymetrix Expression Console software and saved as an ‘.expression’ file containing a unique identifier for each transcript (gene annotation concatenated to probe ID). In subsequent columns, gene and GO annotations were included for assigning class-sets for the analysis of information contained in the network graph, followed by the RMA-normalised raw data. The ‘.expression’ file is subsequently loaded into the network analysis tool BioLayout *Express*^3D^ [30], in which a pairwise Pearson correlation matrix is calculated as a measure of similarity between transcript profiles. A network was created using a threshold of *r ≥* 0.95 and layout performed using modified Fruchterman–Rheingold algorithm [57]. In this paradigm, each node represents a single transcript, which are connected by weighted, undirected edges representing correlations above the threshold. Groups of highly correlated transcripts were then “clustered” using the Markov cluster algorithm [31]. To confirm the changed expression of transcripts between GSS and NBH/101LL brain regions, data were filtered to include only transcripts annotated to known or predicted genes. Each transcript was then tested for statistical significance. As data were not of equal variance, as determined by Kolmogorov–Smirnov test, nonparametric Mann–Whitney *U* test (*p* < 0.05) was performed to assess the change of each transcript intensity in a GSS/101LL brain region compared to its respective NBH/101LL control brain region. Data were then further filtered to include only those transcripts that showed a >1.5-fold change. Further analyses were performed to include a “total” gene list for each GSS/101LL brain region using the same statistical parameters outlined above. Genes in the resulting lists were then assigned likely cell types of origin by inspecting their profile on an expression atlas of cell types on published microarray analyses [32,33], including various types neuronal, glial, and myeloid cell populations. These data were loaded into BioLayout *Express*^3D^ at a Pearson correlation cut-off of (*r ≥* 0.7), and genes were assigned a putative cell type of origin based upon inspection of their expression pattern across the cell atlas. Genes attributable to a microglia origin up-regulated in all regions, regardless of the presence of pathology, were subjected to GO enrichment analysis.

All the primary microarray data have been deposited in GEO: GSE74079.

## Supporting Information

S1 ARRIVE checklist Supporting ARRIVE guidelines checklist. Reporting of animal experiments, which adhere to guidelines established for transparent and reproducibility of experimental data.
(PDF)

S1 Data Dataset with raw data used in Fig 1B. (XLSX)

S2 Data Dataset with raw data used in Fig 2A and 2B. (XLSX)

S3 Data Dataset with raw data used in Fig 4E and 4F. (XLSX)

S4 Data Dataset with raw data used in Fig 5A and 5B. (XLSX)

S5 Data Dataset with raw data used in Fig 6B. (XLSX)

S6 Data Dataset with raw data used in Fig 7A and 7B. (XLSX)

S7 Data Dataset with raw data used in S2 Fig. (XLSX)

S1 Fig PMCA confirms widespread detection of misfolded prion seeds beyond levels detected using IHC. In vitro amplification potential of different brain regions from animals inoculated with GSS and NBH controls (PMCA). GSS/101LL and the NBH/101LL seeds were diluted 1:3 in fresh Tg-P101L substrate. (F) corresponds to the non-sonicated samples and (S) to the sonicated (amplified) samples. vCJD (1/100) is used as a positive PMCA reaction control, and unseeded reactions are used as negative controls. Molecular mass of electrophoretic markers is given in kilodaltons (kDa).
(TIF)

S2 Fig Vacuolation quantification profile of GSS/101LL brain regions at clinical stage pathology. Vacuolation profile of GSS/101LL clinical stage animals (*n* = 9). Grey matter scores (medulla [brain stem], cerebellum, superior colliculus [midbrain], hypothalamus, thalamus, hippocampus, septum, retrospinal cortex, and cingulate and motor cortices) are scored blind on a scale of 0–5, whereby 5 represents severe vaculation and 0 represents no vacuolation.
(TIF)

S1 Table The data in this table have been constructed by assessing the gene expression changes at clinical stages of disease (291.11 ± 5.29 dpi) between GSS/101LL individual brain regions compared to their respective NBH/101LL brain regions. The average (mean) change in gene expression was tested for statistical significance using the Mann–Whitney *U* test, and any data that showed higher than *p* = 0.05 were filtered out of our analysis. Furthermore, we then assessed the fold change of the average gene expression changes, and any data that had a fold change of less than 1.5 were filter out of our analysis. This resulted in "total" lists of differential expressed genes (up- and down-regulated) from each brain region in GSS/101LL mice compared to their respective NBH/101LL controls. In total, this resulted in 3,685 differential regulated genes in brain stem (1,516 up/2,169 down), 1,521 differential regulated genes in thalamus (587 up/934 down), 3,540 differential regulated genes in cerebellum (1,668 up/1,872 down), and 384 differential regulated genes in cortex (112 up/272 down). To further annotate the data, every up-regulated or down-regulated gene identified has been overlaid onto gene expression analysis from isolated cell populations performed in previous studies [32,33]. The overlaid gene lists are then input into BioLayout *Express*^3D^ software set at a Pearson correlation cut-off of r ≥ 0.7. This allows genes to be grouped together based upon their differential expression between cell types isolated in previous studies [32,33]. Once we have accumulated data in BioLayout *Express*^3D^ that can be attributed to specific cell types, we then simplified the nomenclature to fit genes into broad cell type terms. For example, a number of different neuronal cell populations have been assessed by Doyle et al. ranging from Purkinje cells of the cerebellum to cholinergic neurons of the striatum. In this study, however, all genes that can be assigned to any neuronal population have been attributed the cellular origin of neuronal. We also had instances whereby genes would be expressed in cells of both neuronal origin and either astrocytes and/or oligodendrocytes. In these cases, we termed the cellular origin neuronal and glia mix. Please note the following: (i) It is relatively uncommon, but some genes in each list may have duplicates; therefore the numbers of differential regulated genes isn't exact. (ii) The cell attribution data are gathered by groups of genes requiring a relatively high correlation; this means that there will be individual genes whose gene expression data highly correlate with others within the gene lists and therefore are not accounted for in our analysis. In these instances, these genes are assigned N/A, although it is important to note that, despite this, these genes could well be expressed by cell types represented in this analysis. Our analysis offers up a prediction based upon experimental evidence from previous published studies. (iii) Some genes in each gene list have been attributed to be of an "other" cell type. This means that in our analysis, there were groups of genes that showed high correlation with one another, but their gene expression was not indicative of being expressed in any of the cell types onto which we overlapped our data. This does not mean that they are definitely not expressed by any of these cells types, but that we could not assign these genes using the information available to us. This could mean either that their expression belongs to a different cell type present in the CNS or one that invades the CNS during disease or that they are expressed as part of a cellular response mechanism (e.g., autophagy, ER stress, etc.), which was not exhibited in the datasets which we overlaid our data onto.
(XLSX)

S2 Table GO enrichment analysis of genes not directly associated with neurodegeneration. In sheet 1 "GO enrichment," the data presented are constructed by inputting microglial genes that are up-regulated in GSS/101LL cerebellum (see S1 Table) into DAVID. The GO terms were then filtered, firstly for statistical significance by omitting all GO terms with a *p*-value less than 0.03. Next, any GO term with less than a count of ten were omitted from analysis. This resulted in a GO enrichment table of 79 GO terms. A number of common GO terms appeared from the "GO enrichment" sheet, including but not restricted to homeostatic process, immune system process, and metabolic process. However, it was noted that groups of related GO terms (e.g., homeostatic process, cellular homeostasis, chemical homeostasis) shared similar or identical gene lists. Therefore, the genes used to construct each related group of GO terms were plotted into a single gene list, and the replication of genes was then deleted; these data can be viewed in sheet 2 "GO summary." Overall, it was found that almost half of the genes up-regulated in microglia in GSS/101LL cerebellum were related to metabolic process (~31%) or homeostasis (~18%). Please note the following: (i) many of the genes (~22%) that were shown to be up-regulated in microglia in GSS/101LL cerebellum were not assigned to GO terms and therefore are described as "non-attributed genes," and (ii) there are genes that are assigned under multiple and distinct GO terms, and therefore two separate gene lists in "GO summary" may exhibit some overlapping genes.
(XLSX)

S3 Table GO enrichment analysis of genes directly associated with neurodegeneration. In sheet 1 "GO enrichment," the data presented are constructed by inputting microglial genes that are up-regulated in GSS/101LL brain stem and thalamus but that are not up-regulated in GSS/101LL cerebellum (see S1 Table) into DAVID. The GO terms were then filtered, firstly for statistical significance by omitting all GO terms with a *p*-value less than 0.03. Next, any GO term with less than a count of ten were omitted from analysis. This resulted in a GO enrichment table of 155 GO terms. A number of common GO terms appeared from the "GO enrichment" sheet, including but not restricted to immune system process, cell activation, cell proliferation, and metabolic process. However, it was noted that groups of related GO terms (e.g., immune system process, immune response, positive regulation of immune response) shared similar or identical gene lists. Therefore, the genes used to construct each related group of GO terms were plotted into a single gene list, and the replication of genes was then deleted; these data can be viewed in sheet 2 "GO summary." Overall, it was found that almost half of the genes up-regulated in microglia that are associated with neurodegeneration were related to metabolic process (~26%) or immune activation (~21%). Please note the following: (i) many of the genes (~19%) that were shown to be up-regulated were not assigned to GO terms and therefore are described as "non-attributed genes" and (ii) there are genes that are assigned under multiple and distinct GO terms, and therefore two separate gene lists in "GO summary" may exhibit some overlapping genes.
(XLSX)

## Abbreviations

101LL: transgenic 129/Ola mouse with proline to leucine mutation at codon 101 of the prion protein gene
Aβ: Amyloid-beta
dpi: Days post inoculation
GFAP: glial fibrillary acidic protein
GO: Gene ontology
GSS: Gerstmann-Sträussler-Scheinker
Iba1: ionised calcium-binding adapter molecule 1
i.c.: intracerebral
IHC: Immunohistochemistry
KO: Knockout
MAP2: microtubule associated protein 2
NBH: Normal brain homogenate
PK: Proteinase K
PMCA: protein misfolded cyclic amplification
PrP: Prion protein
RT-QuIC: real-time quaking-induced conversion
SN: substantia nigra
SNc: substantia nigra, pars compacta
ThT: Thioflavin-T
TSE: Transmissible Spongiform Encephalopathies
