# Supplementary figures and images for: Distribution of Misfolded Prion Protein Seeding Activity Alone Does Not Predict Regions of Neurodegeneration
Source: PLoS Biol. 2016 Nov 23;14(11):e1002579. doi: 10.1371/journal.pbio.1002579 (PMC5120774; doi:10.1371/journal.pbio.1002579)

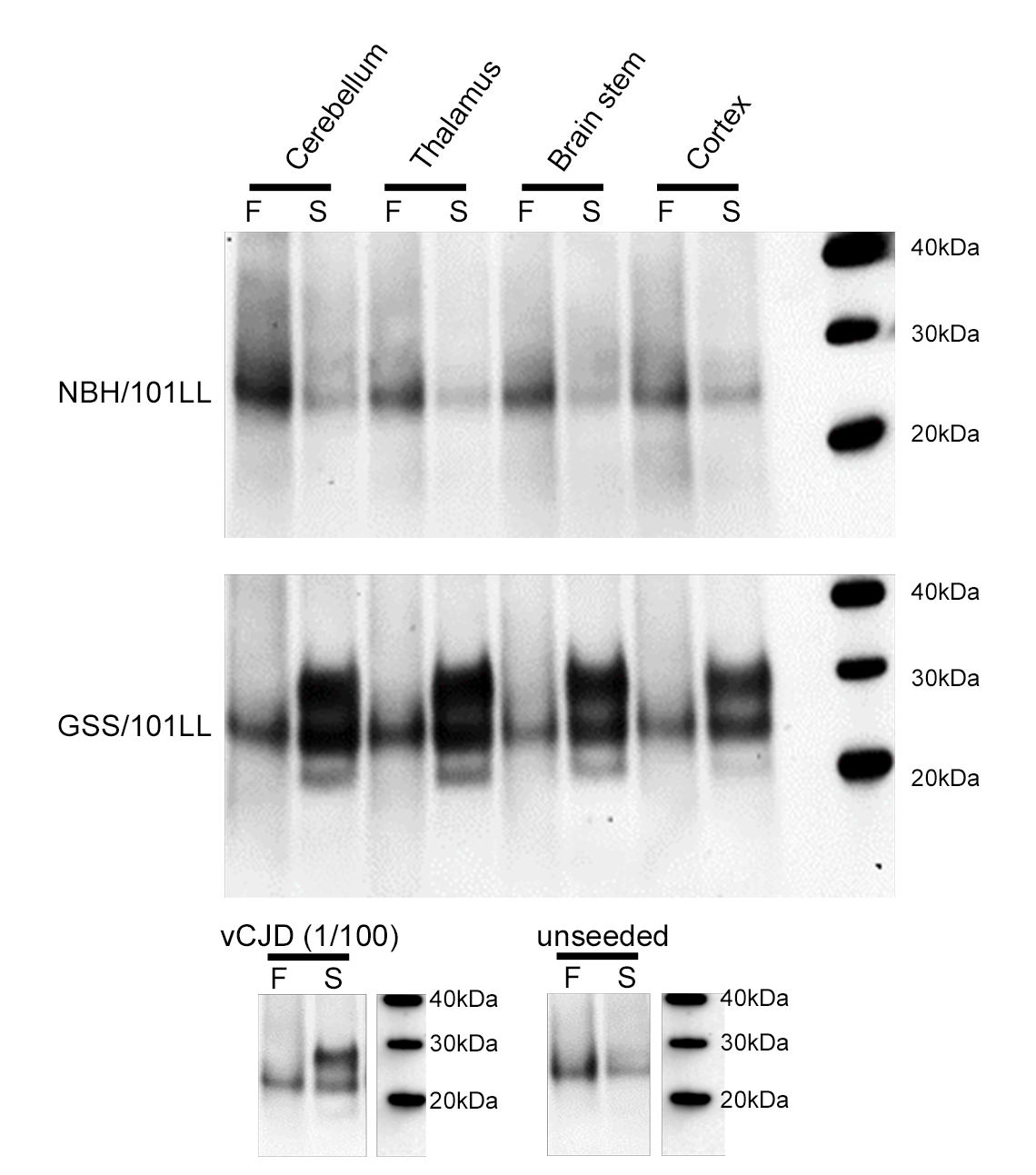

Supplement: S1 Fig — In vitro amplification potential of different brain regions from animals inoculated with GSS and NBH controls (PMCA). GSS/101LL and the NBH/101LL seeds were diluted 1:3 in fresh Tg-P101L substrate. (F) corresponds to the non-sonicated samples and (S) to the sonicated (amplified) samples. vCJD (1/100) is used as a positive PMCA reaction control, and unseeded reactions are used as negative controls. Molecular mass of electrophoretic markers is given in kilodaltons (kDa). (TIF) [file pbio.1002579.s009.tif]

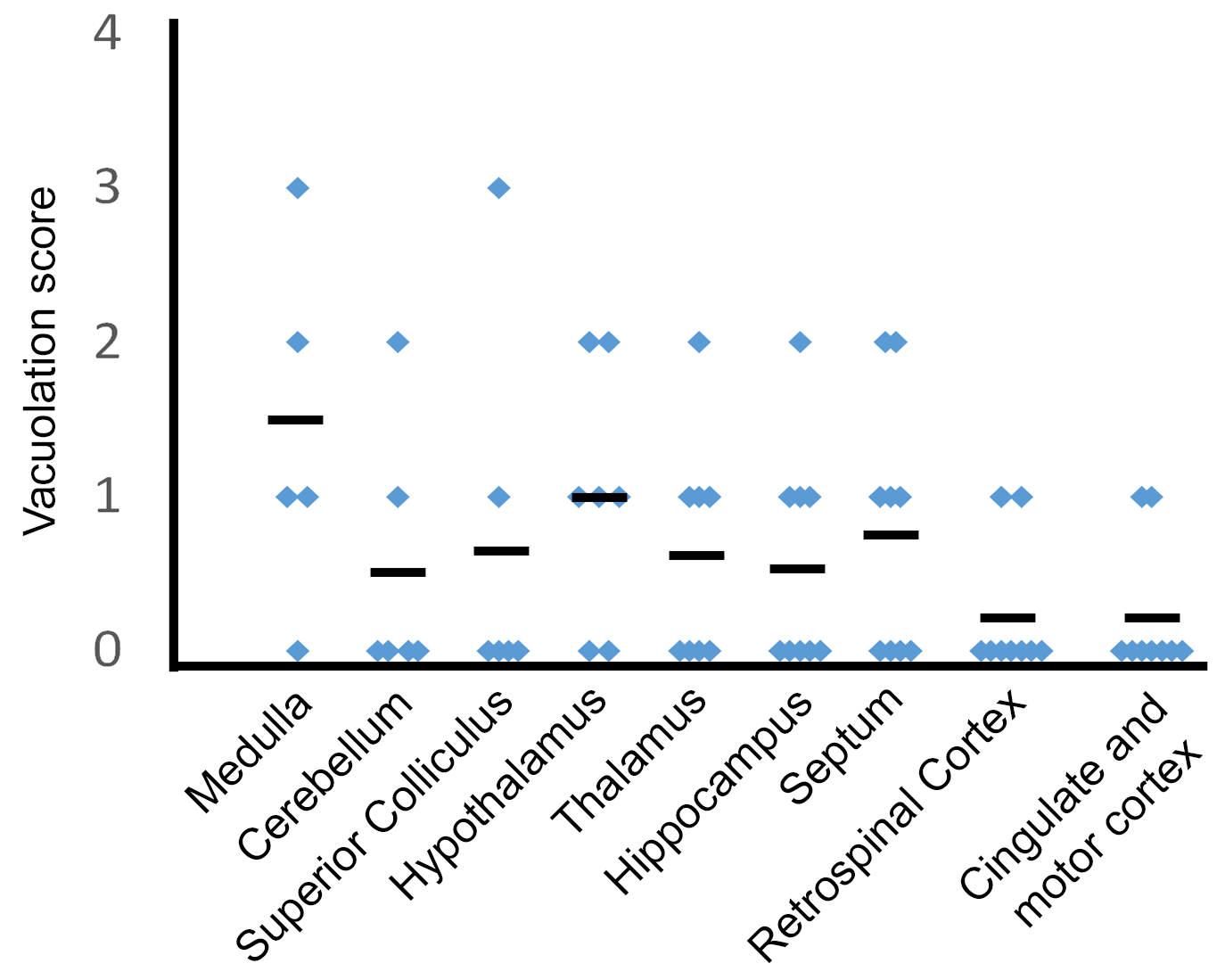

Supplement: S2 Fig — Vacuolation profile of GSS/101LL clinical stage animals (n = 9). Grey matter scores (medulla [brain stem], cerebellum, superior colliculus [midbrain], hypothalamus, thalamus, hippocampus, septum, retrospinal cortex, and cingulate and motor cortices) are scored blind on a scale of 0–5, whereby 5 represents severe vaculation and 0 represents no vacuolation. (TIF) [file pbio.1002579.s010.tif]
